# Impact of KRAS mutation on the tumor microenvironment in colorectal cancer

**DOI:** 10.7150/ijbs.88779

**Published:** 2024-03-03

**Authors:** Yiru Zhou, Yeye Kuang, Chan Wang, Yijian Yu, Lijuan Pan, Xiaotong Hu

**Affiliations:** 1 Biomedical Research Center and Key Laboratory of Biotherapy of Zhejiang Province, Sir Run Run Shaw Hospital, Zhejiang University, Hangzhou, Zhejiang, China.; 2 Department of Pathology, Sir Run Run Shaw Hospital, Zhejiang University, Hangzhou, Zhejiang, China.; 3 Taizhou Hospital of Zhejiang Province affiliated to Wenzhou Medical University, Taizhou, Zhejiang, China.; 4 School of Pharmacy, Hangzhou Normal University, Hangzhou, Zhejiang, China.; 5 Key Laboratory of Elemene Class Anti-Cancer Chinese Medicines; Engineering Laboratory of Development and Application of Traditional Chinese Medicines; Collaborative Innovation Center of Traditional Chinese Medicines of Zhejiang Province, Hangzhou Normal University, Hangzhou, Zhejiang, China.

**Keywords:** Colorectal cancer, KRAS mutation, Tumor microenvironment, Immunotherapy

## Abstract

Kirsten rat sarcoma viral oncogene homolog (KRAS) is an oncogene implicated in the pathophysiology of many cancers. Increasing evidence shows that KRAS mutation is correlated with poor prognosis in numerous cancers, including colorectal cancer (CRC), breast cancer, and melanoma. KRAS also participates in regulating the CRC microenvironment. However, the direct and indirect therapeutic targets of KRAS in CRC have not been identified; thus, elucidating the mechanisms and interactions between KRAS and the tumor microenvironment (TME) in-depth is paramount. Herein, we present some of the major roles KRAS plays in shaping the heterogeneity of the TME and propose a potential strategy for targeting the downstream components of the KRAS signaling pathway and the TME in CRC.

## Introduction

KRAS is one of the most frequently mutated oncogenes across malignancies. KRAS mutations are present in approximately 40% of colorectal cancer (CRC) patients 1. Phosphorylated and dephosphorylated oncogenic KRAS is essential for maintaining the epithelial structure of CRC cells in 3D culture and assisting in the hypodermic growth of tumors in mice 2. Patients with metastatic CRC (mCRC) with KRAS and BRAF mutations have worse progression-free survival (PFS) and overall survival (OS) outcomes than those without these mutations 3. CRC patients with KRAS mutations and liver metastases have more micrometastases, potentially resulting in recurrence after surgery 4. KRAS mutation, which is more common in right-sided colon cancer (RCC) than in left-sided colon cancer (LCC), is correlated with a negative prognosis in LCC patients but not in RCC patients 5. In addition, KRAS mutation is positively correlated with the TMEM16A protein, a calcium-activated chloride channel associated with tumorigenesis and progression in various cancers 6. However, patients harboring multiple KRAS mutations have better prognoses than those harboring a single mutation 7. Nevertheless, enriched KRAS signaling gene sets have been linked to inflammation, a favorable tumor immune microenvironment, and improved survival in triple-negative breast cancer 8.

The rates of KRAS mutation in codons 12, 13, and 61 are 73.3% (11/15), 20% (3/15), and 6.67% (1/15), respectively 9. KRAS G12C is the most prevalent driver mutation and is found in a sizable proportion of CRC patients 10. KRAS G12D is associated with poor survival outcomes and is a marker of poor prognosis in Chinese patients 11. Fortunately, effective and potent G12D inhibitors have recently been developed 12. The difference between G12D and G13D mutations lies in the activation of different signaling molecules 13.

In this review, we first present the pathophysiology and treatment of CRC. The role of KRAS mutation in the development and progression of CRC is then discussed. We also address the impact of KRAS mutation on the TME, including alterations in the immune response, metabolism, and angiogenesis.

## Pathophysiology of CRC

CRC is one of the deadliest diseases worldwide and has serious implications for families and society. An estimated 1.2 million people are diagnosed with CRC annually, and the incidence rate is higher in men than in women 14. The 5-year survival rate has remained at 64% over the past 10 years, and the overall cure rate of CRC has not increased 15. Diet is an important factor affecting the incidence rate; those with a diet containing fiber, milk, calcium, or whole grain have a lower risk of CRC than those with a diet containing processed and red meat 16. A person's lifestyle can be improved by following Mediterranean diet principles to help prevent cancer and other gut-related disorders 17. Polyunsaturated fatty acids may be important in the development of G>A transitions in the KRAS oncogene, potentially contributing to the etiology of CRC 18. In addition, smokers are prone to CpG island methylator phenotype (CIMP)-positive (several CpG island sites simultaneously hypermethylated in CRC) and high-MSI (deficient in mismatch repair system) CRC subtypes 19. A particular cholesterol metabolic abnormality, a potential therapeutic target, was recently discovered in the liver metastases of CRC patients 20. Typically, there are four consensus molecular subtypes (CMSs) of CRC: (1) CMS1 features immune infiltration and mismatch repair mutations. (2) CMS2 cells display the hallmarks WNT and MYC. (3) CMS3 is characterized by KRAS mutation and alterations in metabolism. (4) CMS4 is enriched in the stroma and is associated with the worst prognosis 21. The TME in obese patients promotes the transition of CMS2 CRC epithelial cells toward the CMS4 mesenchymal subtype at the transcriptomic expression level 22.

Multiple KRAS-mutant alleles contribute to the heterogeneous landscape of the TME in KRAS-mutant CRC. Herein, we summarize several features identified by multiomics studies. Based on integrated multiomics data, Chong et al. reported that the KRAS-M1 (KM1) subtype was related to the CMS4 subtype, which, as opposed to the KRAS-M2 (KM2) subtype, is associated with a poor prognosis and increased angiogenesis, EMT, and TGF-β pathways 23. Liu et al. integrated drug sensitivity information and the phosphoproteome and reported that the combination of SHP2 and DOT1L inhibitors is an effective treatment for patients with subset 2 KRAS-mutant cancers 24. Using liquid chromatography-mass spectrometry (LC‒MS/MS) and enzyme-linked immunosorbent assay (ELISA), common methods for proteomic analysis, Lim et al. found that APOPA1 continually increased in the serum of CRC patients compared with that in healthy controls; thus, APOPA1 could serve as a potential biomarker for CRC patients 25. A study of the proteome of the intermediate-stage colorectal cancer cell line Caco2 revealed that the mutant KRAS (V12) contributed to an increased H-Ras protein level, indicating that HRAS might be a pivotal factor involved in the effects of KRAS mutation 26.

At present, the first-line treatments for CRC are surgery, chemotherapy, immunotherapy, and targeted therapy. Current approaches, such as anti-epidermal growth factor receptor (EGFR)-targeted therapies and nonspecific cytotoxic chemotherapeutic regimens, have shown little benefit. Immune checkpoint blockade can be used to treat cancer effectively since glycolysis in cancer cells can be stimulated by interactions between programmed death ligand 1 (PD-L1) on murine CRC cells and PD1 on T cells, increasing effector T-cell access to glucose 27. Regarding targeted therapy, it is extremely difficult to inhibit KRAS directly due to the limited number of available KRAS protein pockets 28. The efficacy of indirect KRAS inhibition is also limited by other cancer-related cellular processes, such as cell Warburg metabolism, a process in which cancer cells preferentially use glycolysis to consume glucose and generate lactate despite the presence of oxygen; this process can be triggered by the mutant KRAS protein and maintain tumor growth 29. The direct inhibitory effects of KRAS mainly include covalent binding to a residue of the protein and blocking the interaction between Ras and its ligands. When KRAS G12C is GDP-bound, these substances covalently attach to the mutant cysteine residue and occupy a novel allosteric pocket 30. Strategies targeting KRAS indirectly include 1) inhibiting the nucleotide exchange cycle, 2) disrupting membrane localization and KRAS processing, and 3) inhibiting downstream signaling pathways. Here, we list several clinical trials to evaluate approaches that directly or indirectly target KRAS. MCLA-158, an LGR5 EGFR bispecific antibody, has therapeutic efficacy in preclinical models of various epithelial cancer types by inhibiting the growth of KRAS-mutant colorectal tumors, blocking metastasis initiation, and suppressing tumor expansion 31. The novel therapies available for KRAS-mutant CRC include sotorasib 32, beta-elemene, cetuximab 33, and adagrasib (MRTX849) 34. Target-specific therapeutic alternatives are needed because most of these clinically approved conventional regimens are frequently accompanied by dose-related toxicity, drug resistance, and unfavorable physiological side effects 35. Another obstacle in CRC treatment is the disease's resistance to epidermal growth factor receptor (EGFR) inhibition conferred by KRAS mutation. The EGFR signaling pathway is the main cause of resistance to KRAS G12C inhibitors; consequently, EGFR and KRAS G12C should be inhibited together 4. Thus, it is imperative to elucidate the role of KRAS mutation in CRC.

## KRAS signaling pathway in CRC

### KRAS activation and downstream effectors

The KRAS signaling pathway is critical for the regulation of cellular growth, division, and survival. The pathway is activated by KRAS, a small GTP-binding protein. KRAS activation leads to the recruitment and activation of downstream effectors, such as RAF, MEK, and ERK, ultimately resulting in the activation of transcription factors, such as ELK1, leading to the expression of genes that regulate cell proliferation, survival, and angiogenesis.

### KRAS mutations and cancer development

The KRAS signaling pathway is tightly regulated in normal cells but is often hyperactivated in cancer cells due to KRAS gene mutations. This hyperactivation leads to uncontrolled cell proliferation and increased cell survival, ultimately leading to the development and progression of cancer. KRAS signaling pathway inhibition has been a major focus of research in cancer therapy, and drugs targeting different pathway components are currently being evaluated in clinical trials.

### Canonical pathways downstream of KRAS mutations

**Figure 1:**
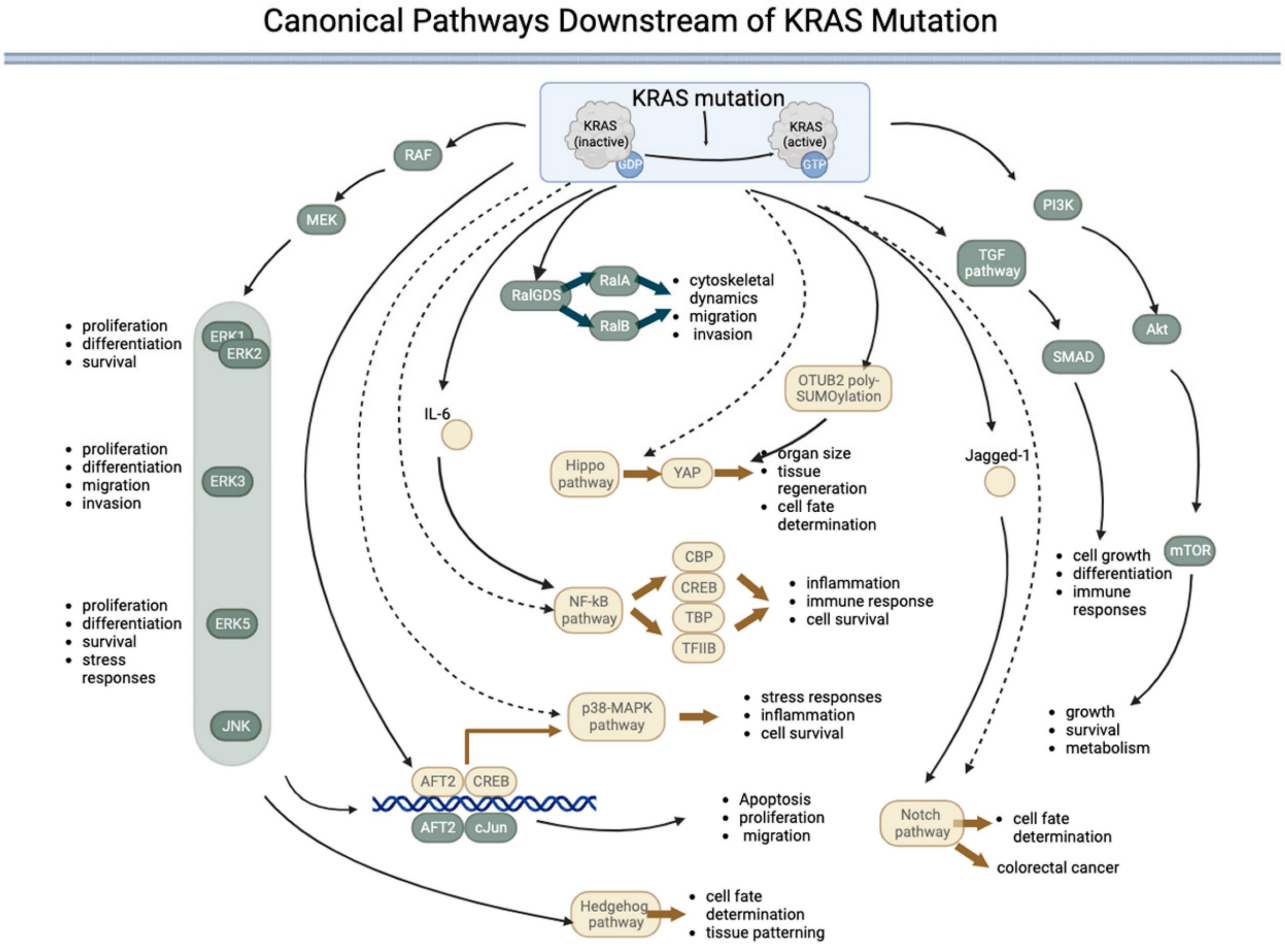
** Canonical pathways downstream of KRAS mutations.** KRAS mutation leads to permanent activation of the KRAS protein, activating several downstream pathways both directly (represented by components in green) and indirectly (represented by components in yellow) regulated by KRAS. Five pathways are directly under the control of KRAS: 1) the RAF-MEK-ERK pathway, 2) the RalGDS pathway, 3) the TGF pathway, 4) the PI3K-AKT-mTOR pathway, and 5) the JNK pathway. The JNK pathway is named after the final kinases (ERK, JNK, and p38) of the RAF-MEK-ERK pathway. The KRAS:1 indirectly regulates five pathways: 1) the p38-MAPK pathway, 2) the NF-kB pathway, 3) the Hippo-YAP pathway, 4) the Notch pathway, and 5) the Hedgehog pathway. (→direct regulated; --> indirectly regulated).

Mutations can permanently activate KRAS, leading to uncontrolled cell growth and proliferation. The downstream signaling pathway of mutated KRAS involves a complex network of proteins and pathways that promote cell proliferation and survival. The main downstream effectors of KRAS are the RAF/MEK/ERK and PI3K/AKT/mTOR pathways. KRAS interacts with multiple signaling pathways, such as the phosphoinositide 3-kinase (PI3K)-protein kinase B (AKT)-mammalian target of rapamycin (mTOR) signaling pathway and the rapidly accelerated fibrosarcoma (RAF)-mitogen-activated protein kinase (MEK)-extracellular regulated protein kinase (ERK) signaling pathway 36. KRAS with the G12D mutation modulates translation via the MNK/eIF4E pathway and results in sustained expression of c-MYC, a common proto-oncogene involved in many cancers 21. KRAS activity is increased by sequence-specific RNA splicing modifications resulting from p53 mutations in pancreatic cancer 37. In addition to the RAF/MEK/ERK and PI3K/AKT/mTOR pathways, KRAS mutations can activate other downstream signaling pathways that promote cell growth and proliferation. The pathways directly activated by KRAS are listed in **Table 1 and Figure 1**.

The RAS-RAF-MEK-ERK signaling pathway is a highly conserved signaling pathway that regulates various cellular processes, including cell proliferation, differentiation, survival, and stress responses 38. The pathway comprises a cascade of kinases sequentially activated downstream of RAS, including RAF, MEK, and ERK proteins (ERK1/2, ERK3, ERK4, and ERK5). Although the core elements of the pathway are similar, there are several key differences between the different ERKs. ERK1 and ERK2, often referred to as canonical ERKs, are the most thoroughly studied members of the ERK family and are ubiquitously expressed. These ERKS regulate numerous cellular processes, including cell proliferation, differentiation, and survival. ERK3, which is less well studied than ERK1 and ERK2, has been implicated in regulating cell proliferation, differentiation, migration, and invasion 42. ERK5 is the most divergent member of the ERK family and is activated by a distinct signaling pathway involving the MEK5 kinase. ERK5 regulates cell survival, migration, and the stress response 43, 44.

The PI3K-AKT-mTOR pathway is activated when KRAS binds to PI3K, leading to the activation of AKT and, subsequently, mTOR. This pathway regulates cell growth, survival, and metabolism 45. The TGF-β pathway regulates cell growth, differentiation, and immune responses. The RAS gene interacts with and regulates TGF-β receptor activity, activating downstream effectors, such as SMAD transcription factors 48, 49. The RalGDS pathway is activated when KRAS binds to RalGDS, activating RalA and RalB 50, 51. This pathway regulates cytoskeletal dynamics, cell migration, and invasion 52, 53. The downstream effectors of KRAS are not always activated in a linear, sequential manner. Instead, there is significant crosstalk between the different pathways, and the activation of one pathway can influence the activity of others. RalGDS is the key component that connects the KRAS pathway to other pathways.

KRAS indirectly activates some pathways, i.e., KRAS interacts with these pathways via an effector. The Notch pathway regulates cell fate determination and differentiation. KRAS can upregulate the expression of Jagged1, a ligand of the Notch pathway, and promote Notch signaling in colorectal cancer cells 54. KRAS's activation of the p38 MAPK pathway can activate transcription factors, such as ATF2 and cAMP response element-binding protein (CREB), and regulate cellular processes, such as stress responses, inflammation, and cell survival 57-61. The p38 MAPK pathway has also been implicated in the development of cancer and other diseases and is a potential therapeutic target. Activation of the JNK pathway by KRAS can lead to the phosphorylation of transcription factors, such as c-Jun and ATF2, which can regulate gene expression and cellular processes, such as apoptosis, proliferation, and migration 62-64. The Hippo pathway regulates organ size, tissue regeneration, and cell fate determination 65. KRAS has been shown to indirectly activate Yes-associated protein (YAP), the downstream effector of the Hippo pathway, by inducing OTUB2 poly-SUMOylation, which can regulate gene expression and cellular proliferation 66. The Hedgehog pathway regulates cell fate determination and tissue patterning during development 67. KRAS has been shown to activate the downstream effector RAF/MEK/MAPK pathway rather than the PI3K-AKT pathway, which can activate the Hedgehog pathway 68. In addition, KRAS has been implicated in regulating the expression of Gli proteins in the KRAS-androgen axis in prostate cancer 69. The NF-κB pathway regulates immune responses, inflammation, and cell survival. Controlled by IL-6, KRAS has been shown to activate the NF-κB pathway during EMT 70, which subsequently regulates downstream effectors, such as transcription factors such as TFIIB, TATA-binding protein (TBP), and CREB binding protein (CBP) 71.

For unknown reasons, each RAS mutation has unique, tissue-specific oncogenic properties. KRAS is an isoform of the Ras family that is ubiquitously expressed in mammals. KRAS mutations are primarily found in pancreatic ductal and colorectal adenocarcinomas 72, whereas NRAS at codon 61 mutations are primarily found in cutaneous malignant melanoma 73. In the GTP hydrolysis cycle, KRAS is in either a GTP-bound active state or a GDP-bound inactive state. KRAS is one of the most common oncogenes in numerous cancers. The three cancers with the highest KRAS mutation rates are pancreatic ductal adenocarcinoma (PDA; > 95%), non-small cell lung cancer (NSCLC), and colorectal carcinoma (CRC; 40%) 74. KRAS gene somatic point mutations account for 30-50% of all CRC cases 36, 75.

The common mechanism of action of the KRAS mutation is glycine substitution with aspartic acid. The wild-type (wt) KRAS protein is momentarily activated after receptor tyrosine kinase (RTK) signal transduction; however, a mutated KRAS protein forces the constitutive activation of downstream signaling pathways, thereby driving a toxic cellular program that is tumorigenic and frequently associated with targeted therapy resistance 76, 77. This mutation allows constitutive KRAS protein activation, thus driving pro-growth and anti-apoptotic signaling pathways without stimulation. Among all patients with positive KRAS mutations, 73.3% (11/15), 20% (3/15), and 6.67% (1/15) of the mutations are in codons 12, 13, and 61, respectively 9. The KRAS G12C mutation is the most prevalent driver mutation and is present in a considerable proportion of CRC patients 10. Patients with KRAS G12D mutations have poor survival outcomes, and this mutation is a marker of poor prognosis in Chinese patients 11. Fortunately, effective and potent G12D inhibitors have been developed in recent years 78. The G12D and G13D mutations activate different signaling molecules 13. Due to the intriguing link between KRAS and other signaling pathways, the efficacy of RAF, MEK, or ERK inhibitors alone is generally unsatisfactory; therefore, combining these inhibitors with other MAPK pathway inhibitors is worthy of investigation 1. The regression of cancers resistant to anti-EGFR therapy requires the concurrent inhibition of PI3K, HER2, and MEK 79. KRAS siRNA and p38α siRNA can be used as a combination treatment to suppress genes involved in CRC 80.

## Mutated KRAS and the CRC TME

### Components of the TME

**Figure 2:**
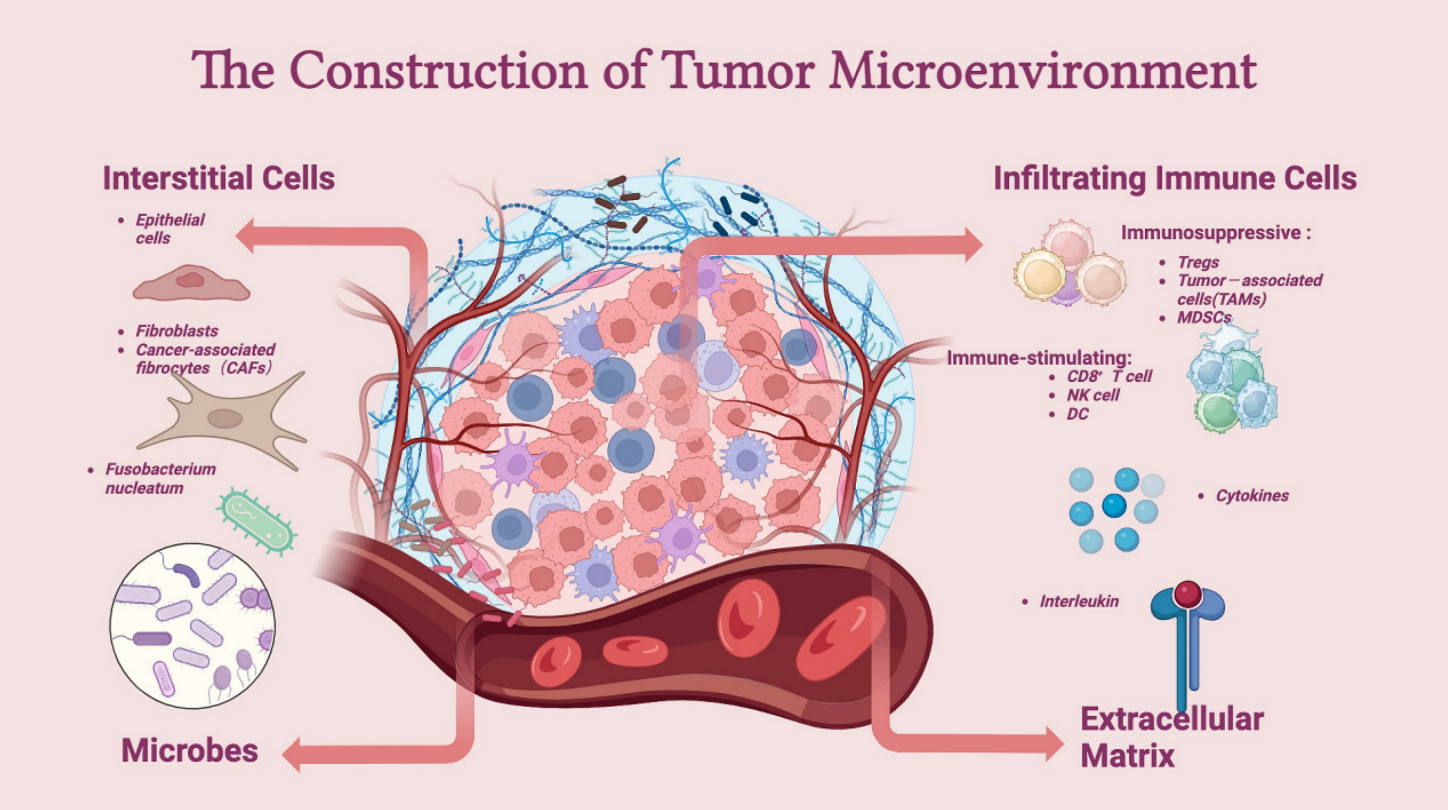
** Construction of the tumor microenvironment.** The tumor microenvironment (TME) mainly consists of interstitial cells, infiltrating immune cells, the extracellular matrix (ECM), and microbes. Immune cells, such as Tregs, TAMs, and MDSCs, are at the core of the establishment of an immune-suppressive microenvironment and can aid tumor cells in escaping the surveillance of the immune system. In addition, interstitial cells, such as CAFs, assist in the growth and metastasis of tumor cells. The extracellular matrix and microbes together influence the dynamics of the TME.

The TME comprises intestinal microbiota, immune cells (such as neutrophils, T lymphocytes, dendritic cells, and macrophages), stromal cells (such as cancer-associated endothelial cells and fibroblasts), and noncellular components (such as the extracellular matrix (ECM)). The TME is essential for regulating or promoting tumor growth. Innate and adaptive immune cells interact with tumor cells to form the TME (**Figure 2**).

The role of T cells in the TME is well established. The ECM supports and connects tissues under normal circumstances and preserves physiologically normal functions 81. An abnormal ECM affects the epithelial-mesenchymal transition (EMT), directly promoting cell transformation and metastasis 82. An important mechanism for the occurrence and growth of CRC is angiogenesis, for which vascular endothelial growth factor (VEGF) is the most important growth factor. Furthermore, CRC cells express three VEGF receptors. Tumor grade, Dukes stage, and lymph node involvement are associated with VEGFR-1, and lymph node involvement is associated with VEGFR-2; however, no clinicopathological variables are associated with VEGFR-3 83. Cancer-associated fibroblasts have been shown to directly secrete exosomes, promoting chemical resistance and CRC metastasis by increasing CRC cell stemness and promoting epithelial-mesenchymal transformation 84. In solid tumor tissues, tumor-associated macrophages (TAMs) are considered the most prevalent immune cell population 85. Through EMT remodeling, TAMs encourage the growth and invasion of colon cancer cells 86. Following infection, the body releases chemokines, causing neutrophils to migrate and detect pathogens 87. An increase in neutrophils in tumors has been linked to a malignant phenotype and can indicate a poor prognosis in CRC patients 88. Tumor-infiltrating lymphocytes (TILs) also play an important role in the TME. Lymphocytes, which include T, B, NK, and NKT cells, are the primary immune cells in tumors. Among these factors, Tregs play an interesting role in the prognosis of cancer. Tregs are a subtype of CD4+ T cells that participate in allergic disease overactivity, immunosuppression, and inflammation 89. However, infiltrating Tregs play different roles in some cancers. In a model of mismatch repair deficiency (dMMR)-CRC, a high level of Foxp3+ Treg infiltration was associated with a higher survival rate 90. However, in a model of lung cancer, Tregs induced metastasis 91. Btla is a coinhibitory receptor that is expressed mainly on B and T cells and is responsible for limiting innate and adaptive immune responses. Btla expressed on Tregs plays a pivotal role in microbial homeostasis. The loss of Btla on lymphocytes is associated with the disruption of microbial homeostasis and elevations in pathogenic and commensal bacteria 92. Decreased CD8+ T-cell effector function in the presence of antigens is related to a dysfunctional immune response in the TME 93. B cells also have antitumor properties as they preferentially localize to the TME 94. However, the role of B cells in the TME is complex. Most studies have shown that the presence of B cells is associated with improved outcomes in cancer patients. However, B cells can generate immunosuppressive cytokines in both animal models and humans, resulting in impaired antitumor immunity and poor clinical outcomes 95.

Many TME cells can produce proinflammatory cytokines (such as TNF-a, IL-1, and IL-6) and certain chemokines, such as CXCL8/IL-8, and the roles of these cytokines and chemokines are related to CRC-associated cachexia 96. Exosomes secreted by tumor and interstitial cells are key components of the TME. The exosome contents include proteins, DNA, miRNA, mRNA, long noncoding RNA, and even virus/prion genetic material 97. SATB2-AS1, a long noncoding RNA, is specifically expressed in colorectal tissues and downregulated in CRC. Survival analyses indicate that decreased SATB2-AS1 expression is associated with poor survival outcomes 98. Functional experiments and bioinformatics analyses revealed that SATB2-AS1 inhibits CRC cell metastasis and regulates TH1-type chemokine expression and immune cell density in CRC 99.

The gut microbial flora plays an important role in the CRC microenvironment. The role of bacteria is complex. Some bacterial families contribute to antitumor immunity, while others promote oncogenesis and progression 100. Microbial dysbiosis can promote colon tumor susceptibility by hyperstimulating CD8+ T cells, leading to early T-cell exhaustion and chronic inflammation, which can jeopardize antitumor immunity 101. The miscellaneous microbiota in neoplasms is also associated with colorectal carcinogenesis 102. One of the potential risk factors for the development and progression of CRC is *Fusobacterium nucleatum* (*F. nucleatum*). The most crucial processes of *F. nucleatum* in CRC are predominantly related to virulence factors, such as FadA and Fap2; microRNAs, such as miR-21, bacteria metabolism, and immunological regulatory components, including myeloid-derived suppressor cells and inhibitory receptors of natural killer cells 103. Toll-like receptors, microRNAs, and autophagy are coordinated by *F. nucleatum* to therapeutically, physiologically, and mechanistically control CRC chemoresistance 104. CRC heterogeneity can be attributed in part to differences in the variety and composition of microbes and their interactions in humans 105. Oral bacterial cancer therapy (BCT) directly affects the tumor epithelium and tumor stem cells 106.

### Pathways involved in the immune system of the TME

**Figure 3:**
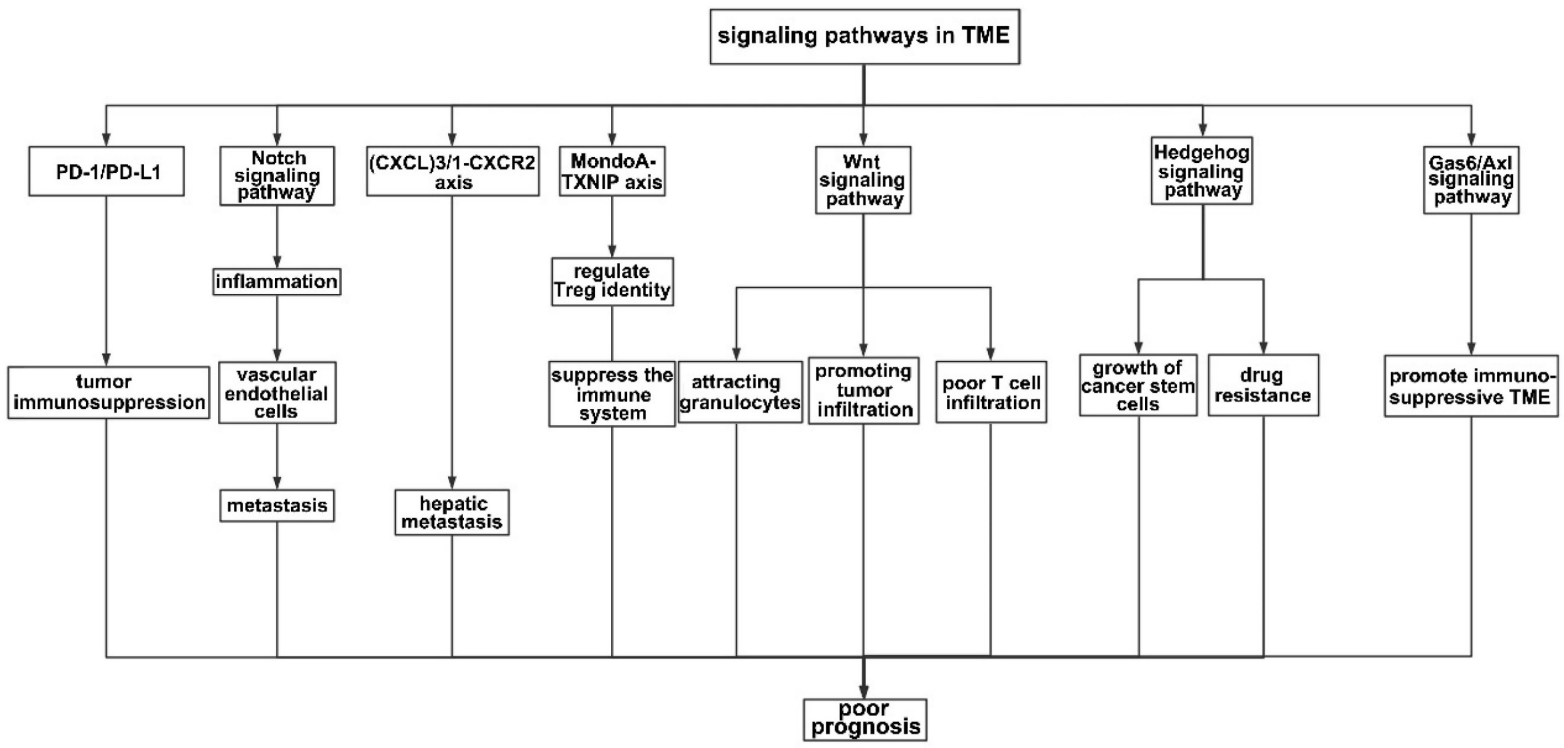
** Signaling pathways in the TME.** PD-1 is an important receptor expressed on the surface of T cells and exerts immunosuppressive effects by prohibiting T cells from reacting excessively. Accordingly, tumor cells produce the corresponding ligand (PD-L1) on their surfaces to prevent T cells from attacking them. In this process, the interaction between PD-1 and PD-L1 leads to immunosuppression in the TME. The Notch signaling pathway mainly affects the integrity of vascular endothelial cells, therefore promoting metastasis. The (CXCL)3-CXCR2 axis promotes hepatic metastasis by attracting immunosuppressive neutrophils to the TME, while the Wnt signaling pathway mainly infiltrates granulocytes. The MondoA-TXNIP axis affects the identity of Treg cells. In addition, HH and the Gas6/Axl pathway participate in the formation of an immunosuppressive TME.

Numerous axes or pathways affecting immune cell interactions are engaged in the tumor environment (**Figure 3**).

The KRAS signaling pathway plays a crucial role in regulating the immune system, from the activation and differentiation of immune cells to the regulation of immune cell apoptosis (i.e., the programmed death of immune cells). The downregulation of the immune system ensures that the immune response is regulated appropriately, preventing excessive inflammation. One of the most important signaling pathways in the TME is the programmed death ligand-1/programmed death-1 (PD-L1/PD-1) signaling pathway, which plays a key role in tumor immunosuppression. However, therapies targeting this pathway remain ineffective.

Inflammatory factors in the TME may be the underlying reason for the failure of anti- PD-L1/PD-1 therapies because inflammation may induce the production of PD-L1, interfering with the efficiency of PD-L1/PD-1 blockade 107. In KRAS G12D-driven serrated cancer, activation of NOTCH1 signaling in the murine intestinal epithelium results in highly penetrant metastasis by creating a TME similar to that of human CRC subtypes with poor prognoses (CMS4 and CRIS-B) 108. Notch activation contributes to metastasis, possibly by influencing vascular endothelial cells. Wieland et al. reported that Notch activation leads to endothelial cell senescence, inducing inflammation and increasing metastasis 109. The chemokine (C-X-C motif) ligand (CXCL) family plays a key role in inflammation, and the level of CXCL is associated with tumor prognosis. Low levels of CXCR1 and CXCR3 are associated with a decreased tumor volume, decreased alpha fetoprotein levels, and a decreased TNM tumor stage 110. The TGF-beta-chemokine (C-X-C motif) ligand (CXCL)3/1-CXCR2 axis can be controlled by KIAA1199, facilitating the infiltration of immunosuppressive neutrophils and contributing to hepatic metastasis 111. The MondoA-TXNIP axis is a crucial metabolic regulator of Treg identity and activity in the microenvironment of CRC and a possible target for cancer therapy 112. In CRC liver metastasis, activation of the Wnt signaling pathway may be crucial for attracting granulocytes and promoting tumor infiltration 113. Poor spontaneous T-cell infiltration is frequently correlated with WNT/β-catenin signaling activation across most human cancers; thus, combination cancer therapy should block Wnt/β-catenin signaling to increase T-cell infiltration 114. The Hedgehog (HH) signaling pathway participates in inflammation, tumor immune tolerance, tumor growth, and drug resistance in the TME. Both the growth of cancer stem cells (CSCs) and the drug resistance of gastrointestinal tumors can be facilitated by aberrant HH signaling activation 115. The Gas6/Axl signaling pathway has been implicated in promoting the immunosuppressive TME 116. In addition, the KRAS pathway has been implicated in the development of various autoimmune diseases, such as rheumatoid arthritis, systemic lupus erythematosus, and multiple sclerosis. In these diseases, an overactive KRAS signaling pathway leads to a hyperactive immune response and chronic inflammation.

### KRAS mutation participates in metabolic interactions

**Figure 4:**
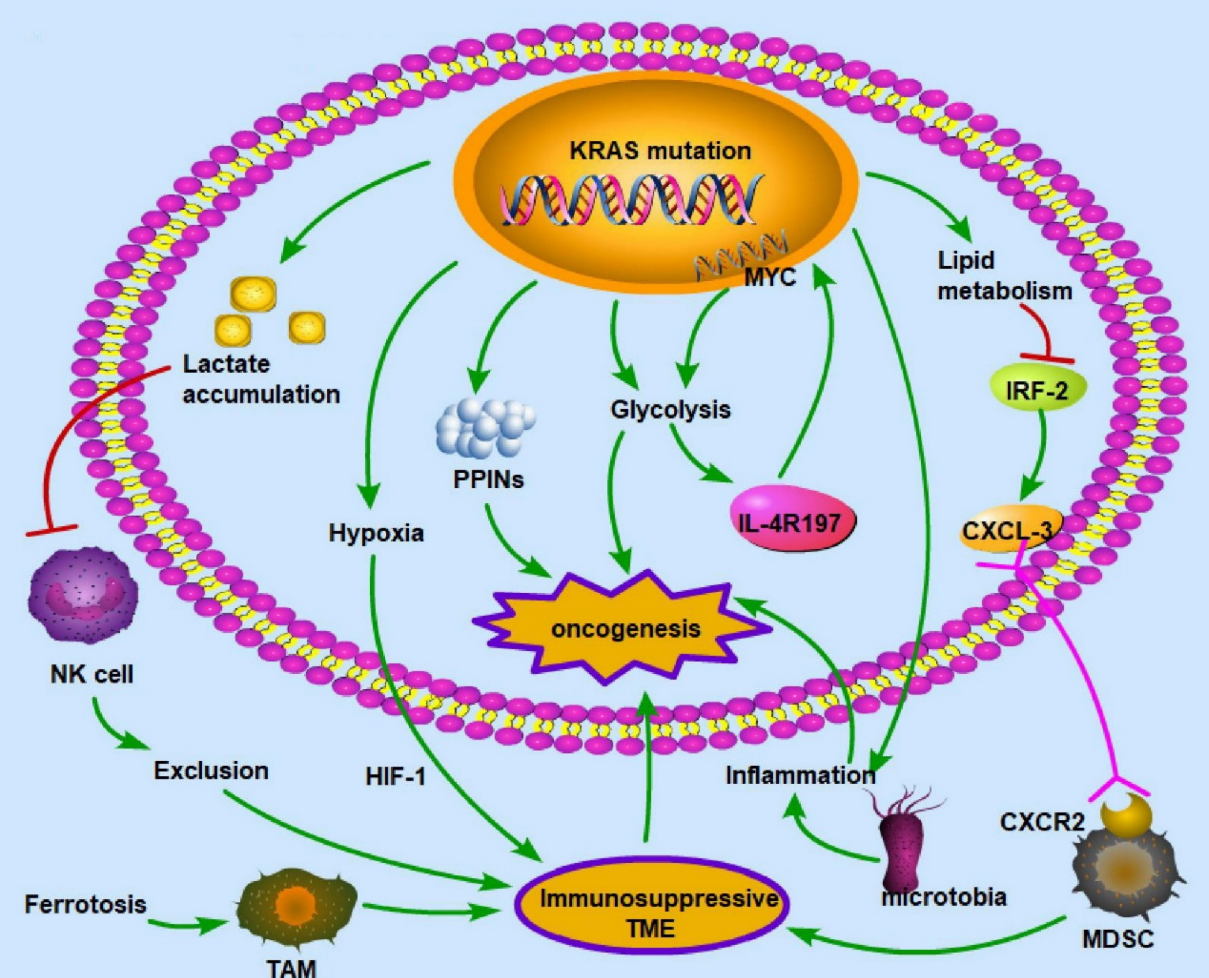
** KRAS mutation-induced metabolic alterations** KRAS mutation can induce oncogenesis by regulating metabolism, including lactate accumulation, hypoxia, PPINs, glycolysis, and lipid metabolism. These alterations influence immune cells and create an immunosuppressive environment, which is necessary for cancer cells to evade immune surveillance. The common patterns for the interaction between metabolism and the immune microenvironment are as follows: exclusion of immune cells with antitumor ability, enhancement of immunosuppressive cells, deprivation of oxygen to immune cells, and inflammation in the microenvironment. Therefore, given its polyfunctionality, mutant KRAS may be a critical target for treating CRC. (→promote; --| inhibit)

KRAS mutation can influence the TME, thus further affecting the prognosis of CRC patients. The oncogenic mutant KRAS controls divergent and convergent metabolic networks in the colon (**Figure 4**).

In the TME, cancer and noncancer cells participate in metabolic interactions by limiting nutrient availability and the activity of the immune system 117. The KRAS signaling pathway in the TME of CRC primarily regulates hypoxia and inflammation, glucose competition, lactate accumulation, amino acid levels, and lipid metabolism 118. For example, the mutated KRAS signaling pathway cooperates with the microbiota in the colon to produce an inflammatory environment conducive to activating oncogenic signaling 119. There is a vicious cycle between KRAS mutation and inflammation in the process of lung tumorigenesis: KRAS mutation can promote inflammation, while enhanced inflammation may also increase the KRAS mutation rate 120. KRAS mutation promotes the autonomous expression of type I cytokine receptor complexes (IL2r-IL4r and IL2r-IL13r1) in cancer cells, which can then bind to and take up cytokine growth signals (IL4 or IL13) released by Th2 cells in the microenvironment 121. Hypoxia is also a key factor in CRC development. In a KRAS-driven mouse model of pancreatic ductal adenocarcinoma, hypoxia results in B-cell exclusion, and it is speculated that the underlying mechanism is that in a hypoxic environment, KRAS mutation results in the exclusion of immune cells via hypoxia-inducible factor-1 122. The hypoxic TME promotes CRC cell stemness and resistance to chemotherapy; thus, this environment could be targeted to mitigate chemoresistance 123. Moreover, glycolysis plays an important role in the immune system. In a mouse model of PDA, KRAS mutation increased the surface expression of IL-4R197, increasing the vulnerability of cancer cells to cytokines produced by TH2 cells 121. This effect increased MYC activity through JAK-STAT signaling and elevated glycolytic gene expression and flux in cancer cells, facilitating tumor growth 121. Furthermore, lactate is a metabolic byproduct of KRAS-mutant metabolism in cancer cells and can create an immunosuppressive TME. Due to a decrease in the intracellular pH and the formation of mitochondrial ROS, lactate in the TME of colon liver metastases causes the apoptosis of infiltrating natural killer cells 124. In the mouse intestinal epithelium, simultaneous mutation of APC and KRAS strongly dysregulates metabolism, enhancing glutamine consumption; thus, the glutamine antiporter SLC7A5 is essential for the development of colorectal tumors 125. KRAS mutation can rewire protein-protein interaction networks (PPINs), altering the cellular phenotype, signal flow, transcriptional regulation, and protein complexes 126.

KRAS mutation can downregulate miR-139-5p and derepress the epithelial-to-mesenchymal transition and oncogenic signaling pathways by disrupting the reciprocal negative feedback mechanism of miR-139-5p/Wnt signaling 127. The KRAS mutation status might be implicated in the development of an oxidative phenotype 128.

### KRAS regulates immune and nonimmune cells (Figure 5)

Immune cells play an important role in the TME. Obesity caused by a high-fat diet inhibits CD8+ T-cell activity in the mouse TME, hastening tumor progression 129. Racial discrepancies in survival outcomes are mediated by the lymphocyte response in the tumor 130. Moreover, nonimmune cells impact the tumor immune microenvironment in CRC 131. For example, chimeric extracellular vesicles are created when tumor cells come into contact with platelets; these vesicles inhibit the growth of the main tumor by activating tumor-eliminating macrophages while promoting metastasis through EMT and endothelial activation 132. Hepatocytes produce various substances that promote cancer cell spreading, attracting or activating stromal cells and immune cells to the liver and creating a favorable premetastatic niche and an immunosuppressive liver milieu conducive to tumor cell colonization and propagation 133. Colon fibroblasts, not tumor cells, are the main cell type that directs infiltrating monocytes toward a particular macrophage population that exhibits high levels of CD163 expression and CCL2 production 134. KRAS can also impact the TME by regulating immune cells, thus affecting the host immune system. KRAS mutations increase the immunogenicity of tumors by increasing the mutational load and hampering DNA repair, thus enhancing neoantigen production 135. KRAS-mediated repression of IRF2 leads to high expression of CXCL3, which binds to CXCR2 on myeloid-derived suppressor cells, thus promoting their migration to the TME 136. Similarly, Treg cells, which play immunosuppressive roles, can also be regulated by KRAS. Mechanistically, mutant KRAS transforms conventional T cells into Treg cells by stimulating the secretion of TGF-beta1 and IL-10 137. The KRAS G12D mutation contributes to regulatory T-cell conversion through activation of the MEK/ERK pathway in pancreatic cancer 138. In a previous study, Tregs were shown to be associated with CD8+ T cell dysfunction and ILC2 upregulation, contributing to KRAS-associated chemoresistance and lymph node metastasis 139. CD8+ T cells, T helper cells, and dendritic cells play crucial roles in the antitumor immune response. Lal et al. analyzed The Cancer Genome Atlas (TGCA) CRC datasets and reported decreased infiltration of cytotoxic cells and a reduced Th1-mediated immune response in patients with KRAS-mutant tumors 140. Moreover, statin-mediated ICD enhances the cross-priming ability of dendritic cells, thereby stimulating CD8+ T-cell immune responses against KRAS mutant tumors 141. The antitumor immune response is also jeopardized through CD47, an antiphagocytosis signal that desensitizes tumor cells to phagocytosis 142. Ferroptosis, which is dependent on autophagy, promotes the polarization of TAMs through the release and uptake of the oncogenic KRAS protein 143. Programmed death ligand 1 translation is suppressed in KRAS G12D tumors, resulting in evasion of immune attack 144. Studies have revealed a greater level of PD-L1 protein expression in KRAS- or TP53-mutant NSCLC than in nonmutant tumors, suggesting that KRAS may participate in immune system inhibition via PD-L1 145. Higher PD-L1 expression enables patients with NSCLC to benefit from immune checkpoint blockade therapy 146. However, another subtype of KRAS mutation (KRAS G12D) was found to be associated with a lower mutation burden in lung adenocarcinoma, resulting in a suppressed immune response 147. In addition, patients with KRAS mutations can also produce a series of cytokines that establish an immunosuppressive microenvironment. In a model of pancreatic cancer, C-C motif chemokine ligand (CCL)15 can be upregulated by KRAS and increase migration and invasion 148. Another C-C motif chemokine ligand (CCL)28 can also be driven by KRAS, which subsequently promotes the upregulation of Fos-like antigen 2 (FOSL2), a novel transcription factor 149. In a KRAS-mutant lung cancer model, the inhibition of CCL5 and interleukin (IL)-6 could hamper cancer growth, suggesting that KRAS mutation may be involved in tumor progression via the production of cytokines 150.

The efficacy of immunotherapy is largely dictated by PD-L1 dysregulation. Zhou et al. reported that dysregulation of PD-L1 induced by UFMylation could result in tumor immune evasion; thus, UFMylation is a potential therapeutic target 151. High PD-L1 or PD-L2 expression is strongly related to poor outcomes, and this dysregulation strongly impacts signaling pathways such as the mTOR, HIF-1, and ERBB pathways 152. T cell-tumor interactions and responses to PD-L1/PD-1 blockade are influenced by molecules expressed on tumor cells, such as CD58 and CMTM6 153. Cytotoxic T cells can be inactivated by PD-L1, leading to restricted immunosurveillance of the TME 154. Therefore, we infer that PD-L1 expression must be fully considered when administering immunotherapy.

### Promotion of angiogenesis by KRAS mutations

Downstream signaling pathways are overactivated when the KRAS gene is mutated, stimulating the production of angiogenic factors and promoting the growth of new blood vessels. This increased angiogenesis is a hallmark of many cancers and contributes to tumor progression, metastasis, and chemotherapy resistance. It is speculated that oncogenes, which are hallmarks of the coagulopathies that occur in many cancers, may also play crucial roles in hemostasis and angiogenesis 155. Activation of the MAPK pathway is associated with upregulated expression of VEGF and improved cell growth and survival in melanoma cells 156. The underlying mechanism of increased VEGF expression might be regulated by activating transcription factors, such as AP-1 (activator protein 1) 157. Endothelial cells outside vascular beds can be stimulated by Ras activation through molecules such as VEGF and COX-derived prostaglandins. In addition, Ras activation can increase uPA/MMP expression and decrease TSP expression, resulting in extracellular matrix remodeling 158. ZNF322A transcriptional activation can also promote neoangiogenesis in lung cancer when KRAS is activated 159.

**Figure 6:**
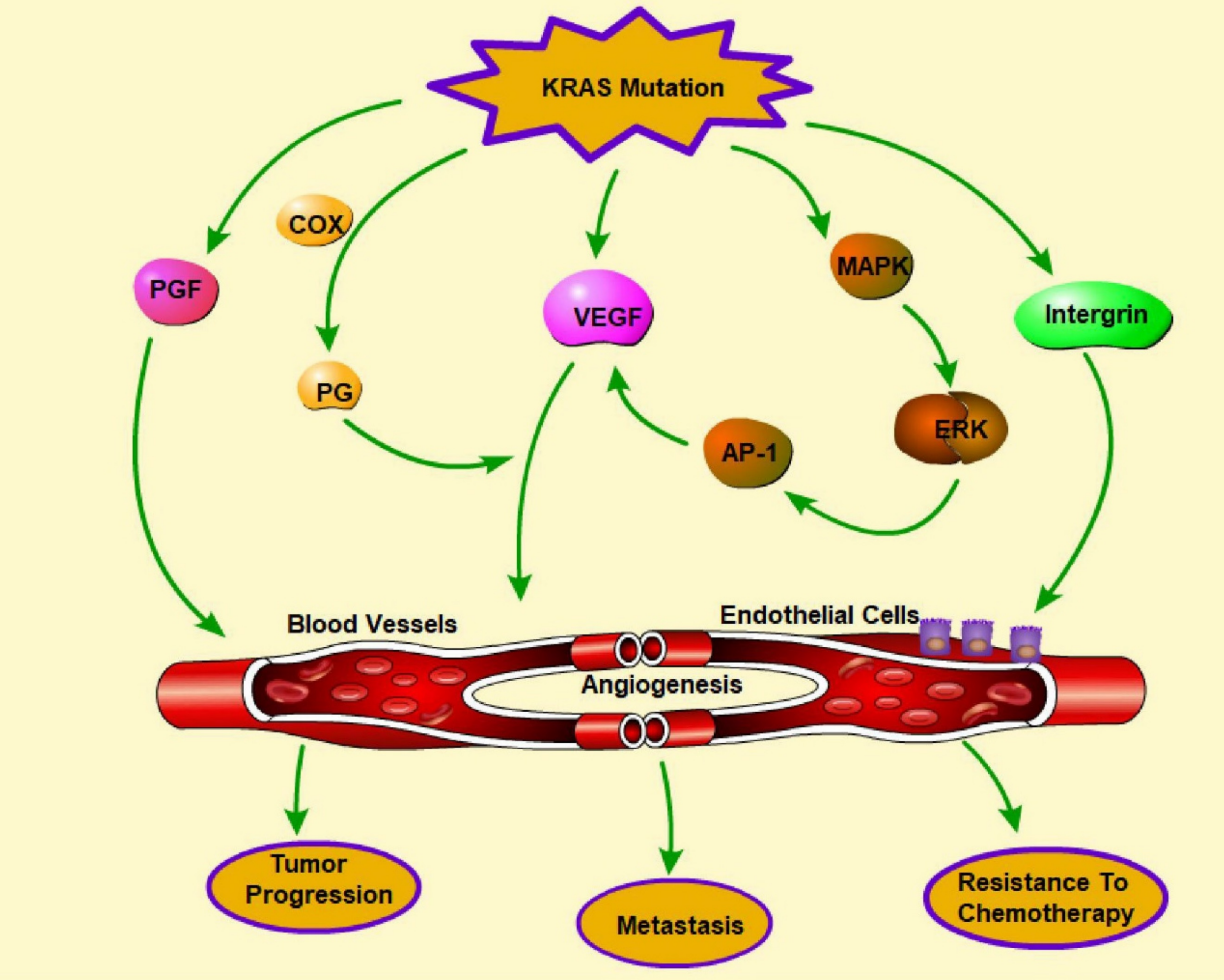
** Regulation of angiogenesis by KRAS mutations.** Angiogenesis plays a pivotal role in tumor progression, metastasis, and chemotherapy resistance. KRAS mutation promotes angiogenesis through the release of multiple molecules, including VEGF, PGF, PG, and integrin, which can influence endothelial cells and the formation of blood vessels. In addition, activation of the MARK-ERK-AP-1 pathway indirectly facilitates angiogenesis by promoting VEGF. (→promote; --| inhibit)

Phenformin, commonly used to treat diabetes, targets extracellular regulated protein kinase (ERK), which is a downstream effector of the KRAS pathway and leads to the inhibition of the expression of proangiogenic molecules 160. ASP13 mutation, which is less harmful than KRAS mutation, can induce a significant VEGF-A-associated vascular network via the RAF-RAS-ERK pathway by activating the VEGF-A promoter when the HIF-1 level is low 161. KRAS mutations can lead to increased fibroblast growth factor expression and activity, resulting in enhanced angiogenesis. KRAS can interact with proteins involved in angiogenesis, such as integrin β4, to promote blood vessel formation, resulting in increased invasion and metastasis 162. Overall, the presence of KRAS mutations in cancer cells can promote angiogenesis, which can lead to aggressive tumor growth and progression (**Figure 6**). Targeting the KRAS signaling pathway and angiogenesis may be a promising strategy for developing new cancer treatments.

## Treatments targeting KRAS and the TME

### Ongoing clinical trials or recently approved drugs targeting the TME

Since KRAS mutations are known to cause many cancers at the genetic level, we now focus on some ongoing clinical trials and recently approved drugs targeting KRAS. Inhibitors can target mutations at different sites. Sotorasib, an inhibitor selectively and irreversibly targeting KRAS (G12C), is used to treat lung cancers caused by the KRAS p.G12C mutation 163. Sotorasib exhibited safe and strong anticancer effects among patients with the KRAS p.G12C mutation who had previously received treatment 164. Adagrasib demonstrates encouraging clinical efficacy and is well tolerated in patients pretreated with KRASG12C-mutated solid tumors 165. Targeting the constitutively active KRAS G12C oncogenic driver with sotorasib and adagrasib has shown promising results, highlighting the need for additional studies to enhance the therapeutic use of these agents in this high-risk population 166. Although sotorasib and adagrasib are currently approved for use in patients with advanced/metastatic NSCLC, regular biomarker testing of KRAS G12C mutations should include testing and reporting before first-line therapy since these tests may aid in clinical decision-making in patients with KRAS G12C-mutated advanced NSCLC 167. In addition to inhibitors that target the G12C mutation (sotorasib and adagrasib), drugs targeting G12D have been developed, such as MRTX1133. Unfortunately, drugs targeting other mutations remain scarce 168.

### Inhibitors and immunotherapies targeting KRAS and its downstream pathways and the TME

A greater comprehension of the effector pathways downstream of KRAS that are either directly or indirectly regulated by KRAS could lead to improved therapeutic interventions. Here, we describe several cutting-edge therapeutic approaches that endeavor to block or hinder the activation of downstream pathways. In KRAS-mutant GC cells, increased expression of DNMT1 was induced by KRAS knockdown, and the combination of a MEK/ERK inhibitor with DNA methyltransferases (DNMTs) might be a promising strategy for GC 169. Dactolisib, a dual PI3K/mTOR inhibitor, can result in greater therapeutic benefits for those with KRAS-mutated lung cancer when combined with Lys05, a dimeric chloroquine 170. Exosomes engineered to promote M1 polarization and inhibit the IL-4 receptor can reprogram TAMs from the M2-like to M1-like phenotype 171. However, macrophage-mediated immune reprogramming and macrophage-mediated drug delivery approaches require substantial optimization 172. Since PI3K-mTOR is the downstream pathway of KRAS, alpelisib, a PI3K inhibitor, was investigated in KRASG12C mutant cancer cells (pancreatic ductal adenocarcinoma 173, ovarian cancer 174, and thyroid cancer 175) and demonstrated synergistic effects with sotorasib, reducing cell viability 176. The development of immunotherapy has improved treatments for several cancers, and it has been shown that the immune system can be trained to identify and eliminate cancer cells. Recently, the use of an enhancer RNA-based subtyping system has shown great potential in prognosis prediction and immunotherapy management in stage II/III CRC patients 177.

Immune checkpoint inhibitors (ICIs) against PD-1 and its ligand PD-L1 effectively treat many cancer patients. However, it is unclear whether KRAS oncogene substitutions impact ICI efficacy. Responses to ICIs that block PD-1/PD-L1 may be highly dependent on concurrent mutations 178. According to several studies, although ICIs can effectively treat patients with gastrointestinal cancers or NSCLC harboring a KRAS mutation, primary or acquired resistance to ICIs may occur 179. Targeted next-generation sequencing of lung adenocarcinoma revealed that KRAS mutation with TP53 and MET mutations is strongly linked to increased PD-L1 expression 180. In KRAS-mutant lung adenocarcinoma, a low TMB and high copy number alteration are potential biomarkers predicting a worse response to ICIs 181. Liu et al. reported that in patients with NSCLC, the KRAS-G12D mutation promoted ICI resistance and immunosuppression, while the combination of chemotherapy and ICI therapy had better efficacy than either treatment alone 182. KRAS mutations cooccurring with TP53 mutations resulted in a notable therapeutic benefit after PD-1 inhibitor administration; therefore, mutations in KRAS and TP53 could be used as predictors for ICI efficacy 119. The mechanism by which KRAS mutation promotes resistance to ICIs may involve the production of immune-suppressive cytokines, such as vascular endothelial growth factor 183, and the suppressive effect on T-cell recruitment 184.

### Role of the microbiome in the treatment of CRC

Microbiomes have been applied in CRC treatment. For example, Tfh-associated antitumor immunity in the colon can be boosted by introducing immunogenic intestinal bacteria, such as *Helicobacter hepaticus* 185. Aryl hydrocarbon receptor activation in tumor-associated macrophages by tryptophan-derived microbial metabolites inhibits antitumor immunity 186. Fecal microbiota transplantation, in which feces from a specific donor are given to a recipient, is a popular therapeutic strategy in cancer treatment. Fecal microbiota transplantation has been used to treat several clinical conditions, including ulcerative colitis, *Clostridium difficile* infection, and other digestive diseases 187. Furthermore, patients who were previously resistant to immune checkpoint inhibitors may respond to therapy after receiving a fecal microbiota transplant 188.

Beta-elemene, a natural product, can induce ferroptosis and prevent epithelial-mesenchymal transformation and can be combined with cetuximab to treat KRAS-mutant CRC 33. Bile acids can also be used to modulate immune cells. Through the activation and recruitment of immune cells that can kill tumors, such as natural killer T cells, bile acids can help promote antitumor immune responses 189.

The butyrophilin molecules BTN2A1 and BTN3A1 can be manipulated to activate Vγ9Vδ2+ T cells, a component of the innate immune system 190. Icariside I improves the microbiota community structure, alleviates inflammation, promotes the restoration of the intestinal barrier, and has significant antitumor effects 191. Characterized by antitumor and anticachexia properties, a ketogenic diet can be used as an adjuvant therapy by reprogramming the epigenome, cell metabolism, and gut microbiome 192.

Atractylenolide I, a component extracted from Rhizoma Atractylodis macrocephalae, may inhibit dysbacteriosis by regulating TLR4/MyD88/NF-κB signaling 193. Alpha-galactosylceramide, produced by bacteria such as *Bacteroides fragilis, Bacteroides vulgatus,* and* Prevotella copri,* exerts antitumor effects by activating nonconventional T cells, such as invariant natural killer T (iNKT) cells and γδ T cells 194. AGPs exert chemopreventive effects by restoring dysbiosis and maintaining enteric homeostasis 195.

### Limitations of current targeted therapies

**Figure 7:**
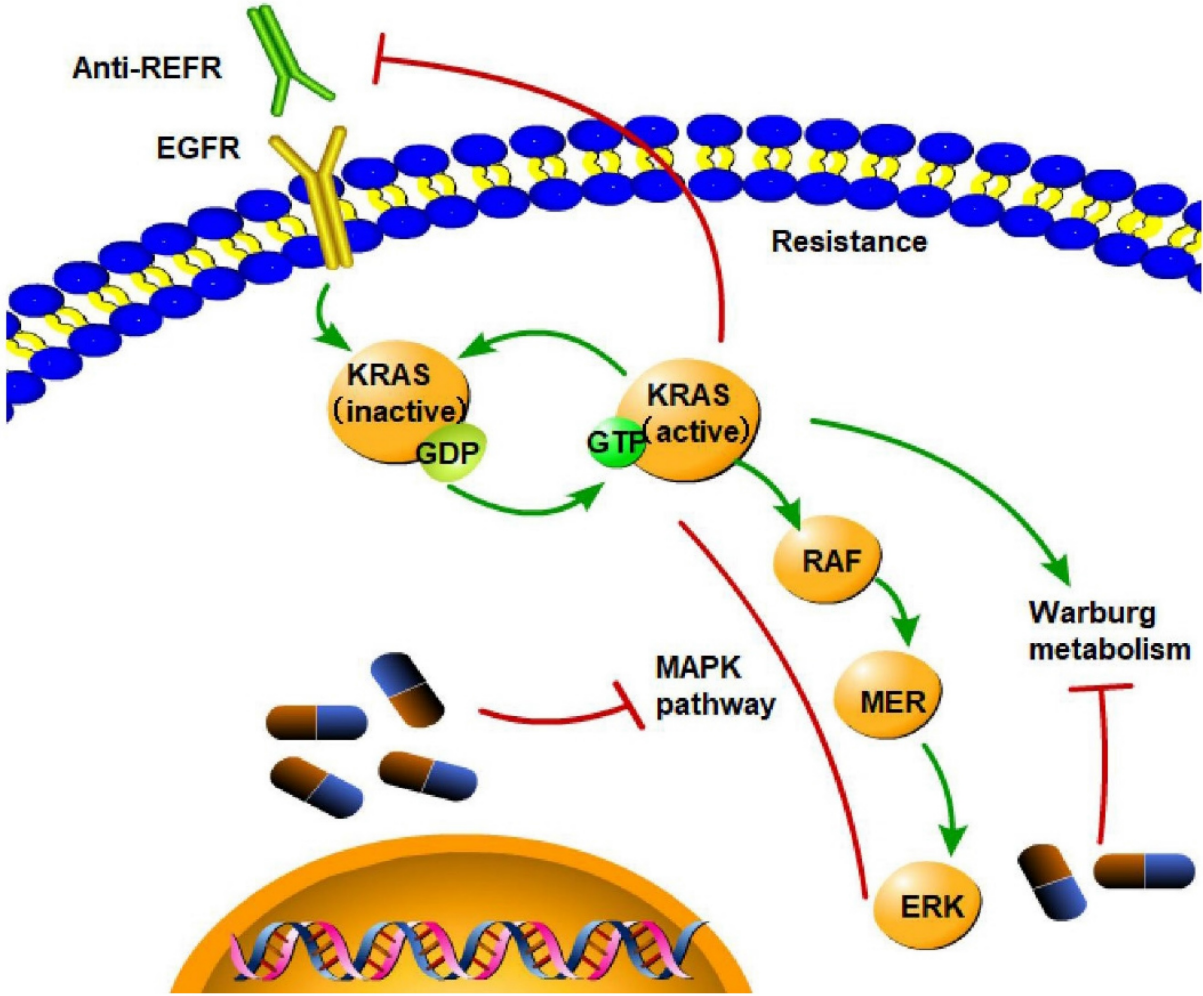
** Targeted treatment of CRC** KRAS mutation is the cause of resistance to anti-EGFR treatment, as it leads to constitutive activation of the MAPK signaling pathway and activation of Warburg metabolism. (→promote; --| inhibit)

Targeting the interactions between mutated KRAS and the TME is an active area of novel therapeutic research aimed at overcoming KRAS-driven resistance to therapy and improving patient outcomes (**Figure 7**).

Since KRAS mutation is related to poor outcomes in CRC patients, KRAS mutation can serve as a target for treating CRC. In-depth research has been conducted using structural and biochemical methods to create KRAS inhibitors. Unfortunately, no specific treatments for KRAS-mutant malignancies have been authorized to date despite the success of small-molecule inhibitor development for other cancer drivers, such as BCR-ABL, C-kit, EGFR, BRAF, and ALK. However, KRAS-mutant CRC is resistant to EGFR inhibitors, and the mechanism is complex. The only reliable biomarkers for determining which patients will benefit from anti-EGFR therapy are skin toxicity, KRAS status, and the European Organization for Research and Treatment of Cancer Classification 196. KRAS mutations are not the primary mechanism of resistance to chemotherapy or anti-EGFR mAbs 197; however, the KRAS G13D mutation is an exception. Cetuximab, an EGFR-blocking antibody, is beneficial for treating KRAS G13D CRC patients 198. In KRAS G12C-mutant NSCLC, epithelial-to-mesenchymal transition causes intrinsic and acquired resistance to KRAS G12C inhibitors 199. The KRAS gene interacts with the KEAP1 gene. Loss of KEAP1 increases NRF2 activity and facilitates KRAS-driven lung adenocarcinoma in mice 200. Thus, it can be inferred that inhibiting the KEAP1 gene might be a potential mechanism for treating CRC. In numerous pancreatic cancer mouse models, the administration of iExosomes that can target oncogenic KRAS-suppressed cancer markedly improves overall survival outcomes 201.

## Conclusion

KRAS mutation plays a crucial role in the development and progression of CRC; therefore, therapies targeting the KRAS gene have become a research focus. Targeted therapeutic drugs that specifically target (directly or indirectly) the KRAS gene, such as sotorasib, adagrasib, and MRTX1133, have been developed and applied in clinical settings. This review summarized current understanding of the mechanism of KRAS mutation and its relationship with the TME. In addition, we promote the potential of targeting KRAS in immune and targeted therapy for CRC by discussing the mechanism underlying the aberrant activation of KRAS signaling coincident with the loss of the antitumor immune responses, including molecular changes in the TME, interactions with the immune system, alterations in metabolism and angiogenesis. Investigating these mechanisms will help develop new therapies targeting these signaling pathways and the TME, improve the prognosis of CRC patients, and identify novel CRC biomarkers.

However, specific and precise targeted therapies are still lacking, and drug resistance and unfavorable physiological side effects remain unresolved issues. In addition, it is difficult to determine which patients are suitable for targeted therapy, and this process requires the use of biomarkers. Consequently, further research should focus on the tumor immune microenvironment and take advantage of the antitumor response to achieve better treatment strategies and reduce resistance to therapy.

## Figures and Tables

**Figure 5:**
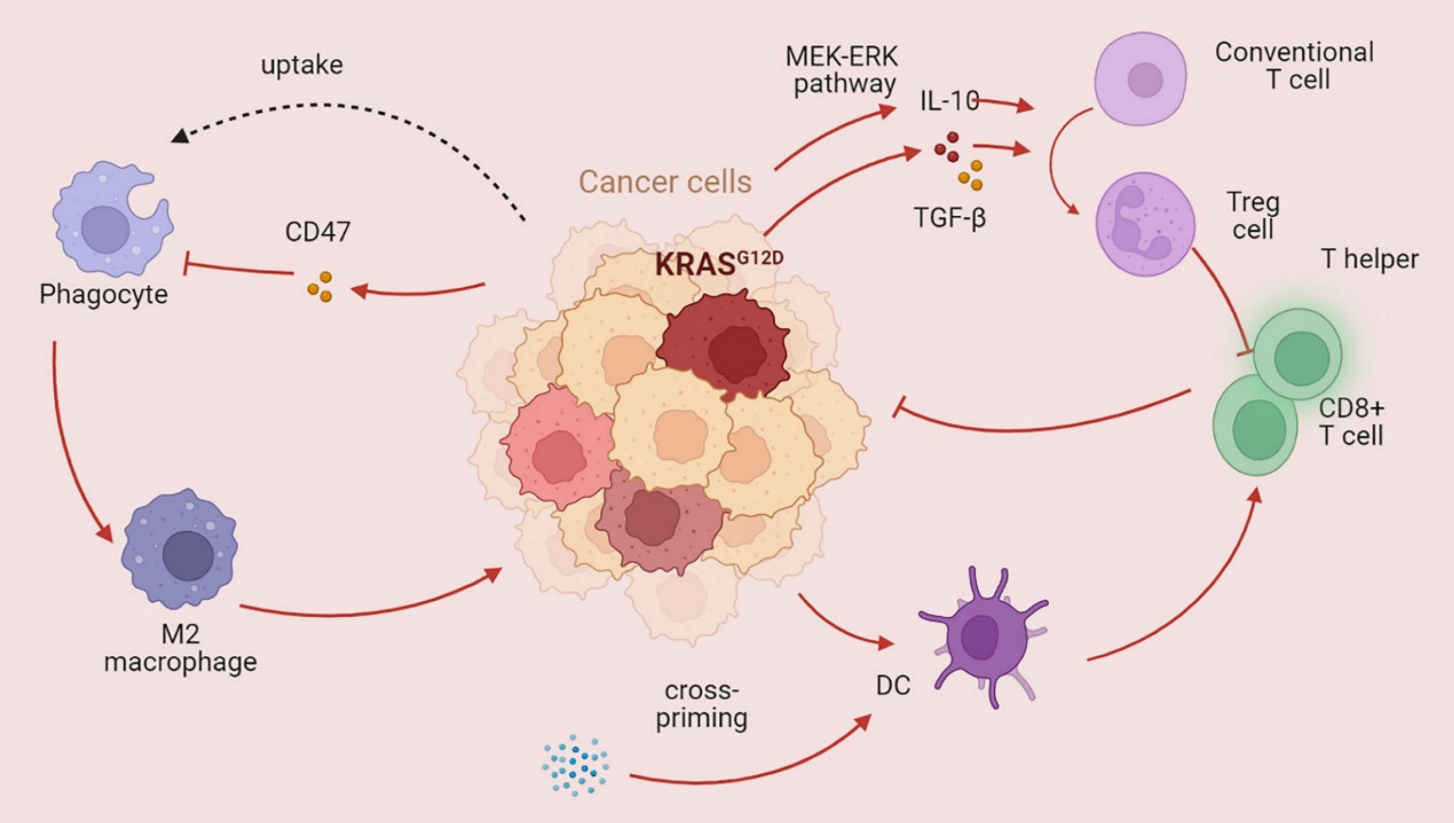
** KRAS mutations affect the tumor immune microenvironment** KRAS mutations play a dual role in the tumor immune microenvironment. The main role of mutant KRAS is destroying the antitumor immune response. KRAS mutation can dampen the function of CD8+ T cells and T helper cells and promote phagocytosis while also promoting immunosuppressive Treg cells (→promote; --| inhibit).

**Table 1:** Canonical pathways downstream of KRAS mutations

Pathway	Directly activated by KRAS?	Downstream/upstream effectors of the pathway	Main role	References
RAS-RAF-MEK-ERK1/2	Yes	AP-1, ELK1, p90RSK, MAPKAPK2, CREB	Regulating cell proliferation, differentiation, and survival.	38-41
RAS-RAF-MEK-ERK3	Yes		Cell proliferation, differentiation, migration, and invasion	42
RAS-RAF-MEK-ERK5	Yes		Survival, migration, proliferation, and differentiation	43, 44
PI3K/Akt	Yes	p70S6K (ribosomal protein S6 kinase) and 4E-BP1 (eukaryotic translation initiation factor 4E-binding protein 1)	Cell growth, survival, and metabolism	45-47
TGF-β	Yes	Cell growth, differentiation, and immune responses	SMAD transcription factors.	48, 49
RalGDS	Yes	RalA and RalB	Cytoskeletal dynamics, cell migration and invasion, and endocytosis.	50-53
Notch	Yes	Jagged1	Regulation of the activity of the transcription factor CSL (CBF1/RBPJk in mammals), a key component of the Notch pathway. Regulation of cell fate determination and differentiation	54-56
p38 MAPK	No	ATF2, CREB (cAMP response element-binding protein),	Stress responses, inflammation, cell survival.	57-61
JNK pathway	No	c-Jun, ATF2	Apoptosis, proliferation, and migration,	62-64
Hippo	No	YAP (Yes-associated protein)	Organ size, tissue regeneration, and cell fate determination,	65, 66
Hedgehog	No	A downstream effector of RAF/MEK/MAPK	Cell fate determination and tissue patterning during development.	67-69
NF-κB	No	IL-6	Regulation of immune responses, inflammation, and cell survival.	70, 71

## Abbreviations

AP-1: Activator protein 1
BCT: Bacterial cancer therapy
CIMP: CpG island methylator phenotype
CMS: Consensus molecular subtypes
CRC: Colorectal cancer
CREB: cAMP response element-binding
CREBP: cAMP response element-binding protein
CSCs: Cancer stem cells
dMMR: mismatch repair deficiency
ECM: Extracellular matrix
EGFR: Epidermal growth factor receptor
EMT: Epithelial-mesenchymal transition
ERK: Extracellular regulated protein kinases
F. nucleatum: Fusobacterium nucleatum
HH: Hedgehog
ICIs: Immunocheckpoint inhibitors
IKK: IκB kinase
iNKT: Invariant natural killer T
KRAS: Kirsten rat sarcoma viral oncogene homolog
LCC: Left-sided colon cancer
MAPK: Mitogen-activated protein kinase
mCRC: Metastatic colorectal cancer
MEK: Mitogen-activated protein kinase
MSI: Microsatellite instability
mTOR: Mammalian target of rapamycin
NSCLC: Non-small cell lung cancer
OS: Overall survival
p70S6K: Ribosomal protein S6 kinase
PDA: Pancreatic ductal adenocarcinoma
PD-L1: Programmed death ligand 1
PFS: Progression-free survival
PI3K: Phosphoinositide 3-kinase
PPINs: Protein-protein interaction networks
RAF: Rapidly accelerated fibrosarcoma
RCC: Right-sided colon cancer
RTK: Receptor tyrosine kinase
TAMs: Tumor-associated macrophages
TILs: Tumor-infiltrating lymphocytes
TME: Tumor microenvironment
VEGF: Vascular endothelial growth factor
wt: wild-type
YAP: Yes-associated protein
4E-BP1: Eukaryotic translation initiation factor 4E-binding protein 1
